# Supplementary material for: Gut microbiome and metabolome profiles in renal allograft rejection from multiomics integration
Source: mSystems. 2025 Apr 24;10(5):e01626-24. doi: 10.1128/msystems.01626-24 (PMC12090775; doi:10.1128/msystems.01626-24)
Supplement: Table S1 — Baseline characteristics of kidney transplant patients. [file msystems.01626-24-s0001.docx]

**TABLE 1** Baseline characteristics of kidney transplant patients

|  | Rejection group  (n=16) | Dysfunction group (n=7) | Control group  (n=12) | *P* value |
| --- | --- | --- | --- | --- |
| Age | 42.4±10.3 | 41.6±11.0 | 35.0±14.2 | 0.131 |
| Sex (M：F) | 13:3 | 5:2 | 10:2 | 0.744 |
| BMI | 26.6±5.0 | 24.2±4.1 | 25.0±3.3 | 0.489 |
| Time after KT (month) | 38.2±23.2 | 43.6±26.0 | 27.3±7.9 | 0.154 |
| UREA | 13.7±4.3 | 9.8±3.7 | 7.8±1.9 | 0.003 |
| CREA | 221.1±57.9 | 121.1±72.1 | 112.0±21.7 | <0.001 |
| UREA/CREA | 0.06±0.01 | 0.06±0.01 | 0.07±0.02 | 0.513 |
| UA | 342.5±63.9 | 346.4±86.6 | 337.3±64.6 | 0.972 |
| UPRO/CREA | 80.0±60.0 | 184.6±225.9 | 24.5±6.1 | 0.017 |
| eGFR | 31.5±8.2 | 38.3±21.7 | 74.5±15.6 | <0.001 |
| HLA mismatch |  |  |  |  |
| HLA A/B mismatch | 2.50±0.89 | 2.14±1.07 | 1.77±0.73 | 0.122 |
| HLA DR mismatch | 1.00±0.52 | 1.29±0.76 | 1.08±0.64 | 0.549 |
| PRA pretransplantation | 2(12.5%) | 1(14.3%) | 1(8.3%） | 0.199 |
| ABO incompatibility | 1(6.25%) | 0(0%) | 3(25%) | 0.173 |
| Intraoperative antibiotic |  |  |  | 0.897 |
| Cefoperazone/Sulbactam | 10(62.5%) | 4(57.1%) | 8(66.7%) |  |
| Piperacillin-Tazobactam | 6 (37.5%) | 3(42.9%) | 4(25%) |  |
| intraoperative induction regimen |  |  |  |  |
| Basiliximab | 16 | 7 | 12 |  |
| postoperative immunosuppressive regimen |  |  |  |  |
| FK+MMF+Pred | 16 | 7 | 12 |  |
| Donor information |  |  |  |  |
| Age | 44.8±11.8 | 42.7±11.5 | 48.5±0.95 | 0.576 |
| Sex mismatch | 3(18.8%) | 1(14.3%) | 4(33.3%) | 0.551 |
| Warm ischemia time | 3.94±2.44 | 3.86±1.22 | 3.00±1.58 | 0.383 |
| Cold ischemia time | 4.13±2.06 | 4.14±1.07 | 3.46±1.71 | 0.594 |
| Type |  |  |  | 0.691 |
| DCD | 2(12.5%) | 2(28.6%) | 2(16.7%) |  |
| Living related donor | 14(87.5%) | 5(71.4%) | 10(83.3%) |  |
| Infection information |  |  |  |  |
| Any infection after transplantation | 5(31.3%) | 2(28.6%) | 3(25.0%) | 0.306 |
| Only CMV | 3 | 1 | 1 |  |
| Only BK viremia | 0 | 0 | 0 |  |
| Only Bacterial | 1 | 0 | 0 |  |
| Only Fungal | 0 | 0 | 1 |  |
| Bacterial and Fungal | 1 | 0 | 0 |  |
| BK viremia and Bacterial | 0 | 1 | 1 |  |
